# Splenic red pulp macrophages provide a niche for CML stem cells and induce therapy resistance

**DOI:** 10.1038/s41375-022-01682-2

**Published:** 2022-09-26

**Authors:** Elias D. Bührer, Michael A. Amrein, Stefan Forster, Stephan Isringhausen, Christian M. Schürch, Salil S. Bhate, Tess Brodie, Joel Zindel, Deborah Stroka, Mohamad Al Sayed, César Nombela-Arrieta, Ramin Radpour, Carsten Riether, Adrian F. Ochsenbein

**Affiliations:** 1 Department of Medical Oncology, Inselspital, Bern University Hospital, University of Bern, Bern, Switzerland; 2 Tumor Immunology, Department for BioMedical Research, University of Bern, Bern, Switzerland; 3 Graduate School of Cellular and Biomedical Sciences, University of Bern, Bern, Switzerland; 4 Department of Medical Oncology and Hematology, University Hospital and University of Zürich, Zürich, Switzerland; 5 Institute of Pathology, University of Bern, Bern, Switzerland; 6 Baxter Laboratory for Stem Cell Biology, Department of Microbiology and Immunology, Stanford University School of Medicine, Stanford, CA USA; 7 Department of Pathology, Stanford University School of Medicine, Stanford, CA USA; 8 Department of Pathology and Neuropathology, University Hospital and Comprehensive Cancer Center Tübingen, Tübingen, Germany; 9 Visceral Surgery, Department of BioMedical Research, University of Bern, Bern, Switzerland

**Keywords:** Cancer stem cells, Stem-cell research

## Abstract

Disease progression and relapse of chronic myeloid leukemia (CML) are caused by therapy resistant leukemia stem cells (LSCs), and cure relies on their eradication. The microenvironment in the bone marrow (BM) is known to contribute to LSC maintenance and resistance. Although leukemic infiltration of the spleen is a hallmark of CML, it is unknown whether spleen cells form a niche that maintains LSCs. Here, we demonstrate that LSCs preferentially accumulate in the spleen and contribute to disease progression. Spleen LSCs were located in the red pulp close to red pulp macrophages (RPM) in CML patients and in a murine CML model. Pharmacologic and genetic depletion of RPM reduced LSCs and decreased their cell cycling activity in the spleen. Gene expression analysis revealed enriched stemness and decreased myeloid lineage differentiation in spleen leukemic stem and progenitor cells (LSPCs). These results demonstrate that splenic RPM form a niche that maintains CML LSCs in a quiescent state, resulting in disease progression and resistance to therapy.

## Introduction

Chronic myeloid leukemia (CML) is a myeloproliferative neoplasm caused by a reciprocal translocation between chromosome 9 and 22, creating the oncogenic fusion gene BCR-ABL1, which encodes for a constitutively active tyrosine kinase [1]. The BCR-ABL1 translocation occurs at the level of hematopoietic stem cells (HSCs), resulting in the generation of a CML initiating leukemia stem cell (LSC). Clinical and experimental evidence suggest that LSCs are resistant against current therapies and responsible for relapse after treatment-induced complete remission [2]. Therefore, definite cure of CML requires the eradication of LSCs [3]. Various mechanisms contribute to the therapy resistance of LSCs, such as drug efflux proteins, newly acquired mutations, and protective signals from the surrounding microenvironment, the so-called niche [4, 5].

The stem cell niche in the BM consists of different cell types that maintain and regulate HSC functions such as quiescence, self-renewal, and lineage commitment [5, 6]. In the case of leukemia, the HSC niche is hijacked by LSCs, which depend on similar signals as their healthy counterparts [7]. Moreover, LSCs can remodel the HSC niche into a malignant niche that is permissive of leukemia growth and disrupts normal hematopoiesis [8]. Overall, the LSC niche in the BM has been extensively studied and several potential therapeutic approaches targeting LSC-BM interactions are under evaluation [9–11].

Splenomegaly is a hallmark of CML and is caused by infiltration of leukemic cells and extramedullary hematopoiesis (EMH). Inra et al. demonstrated that splenic EMH depended on a perisinusoidal niche consisting of endothelial and stromal cells in the red pulp to retain HSCs [12]. In addition, Dutta et al. identified red pulp macrophages (RPMs) as crucial niche components to retain HSCs in the spleen during EMH [13]. Similarly, CML LSCs have been identified in the spleen [14]. However, whether spleen cells form a niche and regulate LSCs has not been studied so far.

Here, we characterized the splenic leukemia stem and progenitor cell (LSPC) niche in CML. We found that the spleen harbored predominantly primitive LSCs, which were quiescent and more resistant to imatinib treatment compared to LSCs from the BM. In addition, leukemic cell differentiation in the spleen was impaired leading to an accumulation of common myeloid progenitors (CMPs). Gene expression analysis of LSPCs revealed an enrichment of stemness genes and a downregulation of genes involved in myeloid lineage commitment in the spleen. LSCs in the spleen exclusively located in the red pulp and were dependent on RPMs. Splenectomy reduced the accumulation of LSCs and prolonged survival of CML mice. Therefore, RPMs in the spleen contribute to disease progression and resistance to TKI therapy in CML by accumulating a large pool of primitive LSCs.

## Material and methods

### Mice

C57BL/6 (BL/6) mice were purchased from Charles River and kept under specific pathogen free conditions. Spi-C loss of function mice [15] were obtained from Prof. Christian Kurts, Rheinische Friedrich-Wilhelms-University, Bonn, Germany. All experiments were performed with sex- and age- matched (6–12 weeks) animals according to Swiss laws for animal protection. No randomization was used for animal studies. When analyzing animal experiments, no blinding was done.

### Splenectomy

Splenectomy was performed as previously published [16] and animals were allowed to recover for at least 2 weeks before CML induction.

### Lineage depletion and flow cytometry

Organs were harvested, flushed (bones) or smashed (spleens), and red cells were lysed. Lineage depletion was performed using biotinylated antibodies against red cell precursors (anti-Ter119), B cells (anti-CD19), T cells (anti-CD3e), and myeloid cells (anti-Gr1), magnetic activated cell sorting (MACS) anti-biotin beads, and an autoMACS pro seperator (Milteny Biotec). Negative and positive separations were stained with fluorescent antibodies and analyzed on a BD LSR Fortessa (BD Biosciences) device or sorted with a BD FACSAria (BD Biosciences). Data were analyzed using FlowJo software (TreeStar).

### CML model

CML was induced in mice by retroviral transduction of BM lin^−^, Sca-1^+^, ckit^+^ (LSK) cells with the vector pMSCV-p210BCR/ABL-IRES-GFP as described before [17]. BM and spleen were analyzed 18 days after transplantation. Endpoint in survival experiments was determined as day 60 after transplantation. In case of weight loss >20%, abnormal gait or posture, or failure to groom, mice were euthanized

### Secondary transplantations

For secondary transplantation experiments, 5 × 10^4^ LSCs from BM or spleen were transplanted into non-irradiated or sublethally (4.5 Gy) irradiated BL/6 recipient mice. LSC frequencies were determined by transplanting 1 × 10^6^, 1 × 10^5^, and 1 × 10^4^ FACS-purified GFP^+^ cells into lethally irradiated (2 × 6.5 Gy) BL/6 recipient mice.

### RPM Isolation and depletion

Spleens were smashed, total splenocytes were loaded on MACS columns and the magnetic positive fraction was stained for F4/80 and CD11b followed by FACS isolation [18].

For pharmacologic RPM depletion, BL/6 CML mice were treated with 1 ml of clodronate liposomes (equal 5 mg of clodronate) (Liposoma, the Netherlands (clodronateliposomes.com)) or vehicle (PBS) every 5^th^ day by intraperitoneal injection starting 3 days prior to CML induction.

### CyTOF analysis

Cells were stained according to standard published methods for mass cytometry [19]. An antibody cocktail for surface and intracellular/intranuclear was used (Table S1). The samples were acquired on a Helios mass cytometer the following day. FCS files were pre-processed using the premessa R package (Parker Institute for Cancer Immunotherapy) for FCS file concatenation and debarcoding. An R graphical user interface is provided for each operation and the edited fcs files are returned. We used the values of 0.2 Minimum separation and 30 Maximum Mahalanobis distance when de-barcoding. FCS files were uploaded into Cytobank and target expression was checked for signal stability over time. We ensured that all targets were clearly stained and we did not observe pressure drops or spikes over time. Clean-up gates were applied to remove beads, antibody aggregates, doublets and dead cells using the Gaussian gating strategy developed by Fluidigm and Verity Software House. Cleaned FCS files were then analyzed by using a standardized Bioconductor workflow in R as previously described [20]. Residual contaminating lymphocytes were gated before clustering based on their CD19 and CD3 expression.

### Statistical analysis

Data was analyzed using GraphPad Prism 7 (GraphPad software). Data are displayed as mean ± SEM. The following tests were used as indicated in the figure legends: two-tailed student’s *t* test, one-way ANOVA, two-way ANOVA, log-rank test, linear regression, ELDA analysis [21]. *P* values < 0.05 were considered significant.

### Study approval

All animal experiments were approved by the veterinary office of the Canton of Bern, Switzerland. Analysis of human samples was approved by the local ethical committee of the Canton of Bern, Switzerland (KEK 122/14 and 2019-01627).

## Results

### The majority of CML-LSPCs accumulates in the spleen

CML in mice was established by retroviral transduction of LSKs with BCR-ABL1 and intravenous transplantation (Fig. S1A) [17]. To prevent irradiation-induced changes in the spleen and BM microenvironment, we transplanted BCR-ABL1-GFP-transduced LSKs into non-irradiated recipient mice. CML developed over a period of 15 to 20 days and showed consistent splenic involvement with splenomegaly (Fig. S1B). LSPCs were identified as previously reported (Fig. S1C) [22]. LSCs first homed to the BM and no GFP^+^ leukemia cells were detectable in the spleen 18 h after injection (Fig. S1D). In contrast, 18 days after CML induction, numbers of LSPC subsets were up to 10-fold higher in spleen than in BM (Fig. S2A-L). This suggests that CML is first initiated in the BM and LSPCs migrate to and accumulate in the spleen during disease progression.

**Fig. 1:**
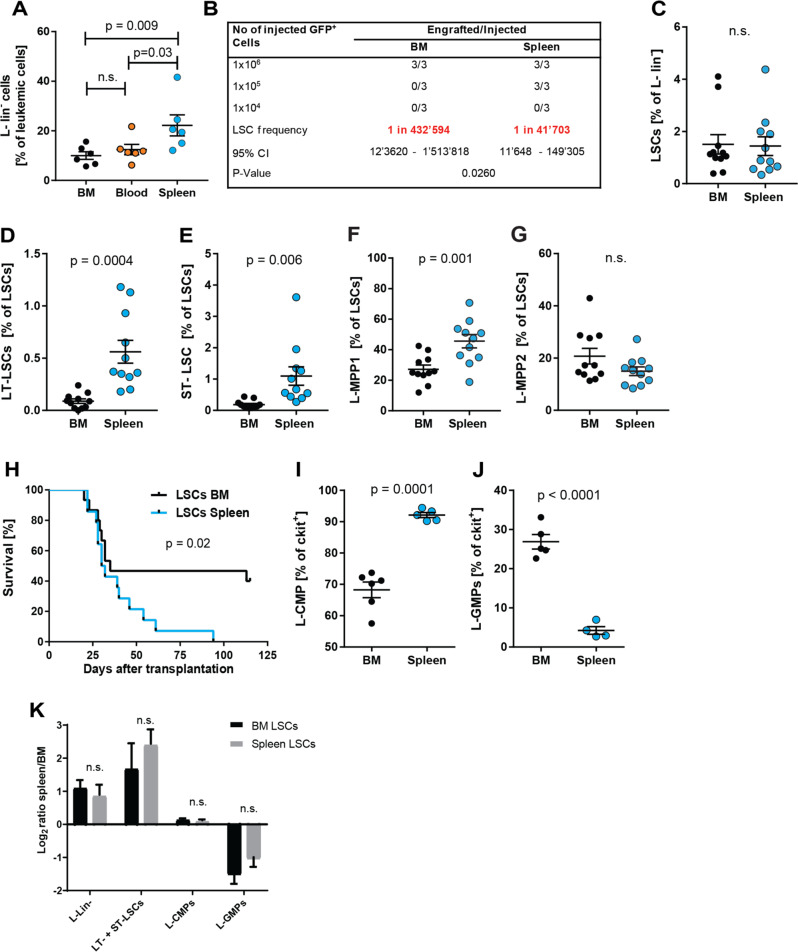
The majority of LSPCs is located in the spleen. **A** L-lin^−^ were analyzed in BM, spleen and blood of BL/6 CML mice 18 days after transplantation. One representative experiment out of 5–10 is shown, *n* = 6 mice per group. **B** Limiting dilution transplantation of BCR-ABL1-GFP^+^ cells from spleen or BM into lethally irradiated BL/6 mice. **C**–**G** Frequencies of LSPC subsets in spleen and BM 18 days after CML induction. One representative experiment out of 5–10 is shown, *n* = 11 mice per group. **H** Kaplan–Meier survival curves of secondary BL/6 CML mice. In total, 2 × 10^4^ FACS purified LSCs from spleen or BM were transplanted into sublethally irradiated BL/6 recipient mice. Data are pooled from two independent experiments with *n* = 13–15 mice per group. **I**–**J** Frequencies of L-progenitor cells in spleen and BM 18 days after CML induction. One representative experiment out of 5–10 is shown, *n* = 5–6 mice per group. **K** In total, 5 × 10^4^ FACS purified LSCs from BM or spleen were secondarily transplanted into non-irradiated BL/6 mice. LSPCs in BM and spleen of secondary recipients were analyzed 24 days after transplantation. The ratio of the frequency of the individual cell population in spleen versus BM is indicated. Pooled data from 2 independent experiments with *n* = 5–10 mice per group is shown. Data are displayed as mean ± SEM. Statistics: Unpaired student’s *t* test (**A**, **C**-6, **I**, **J**), ELDA (**B**), log-rank test (**H**).

Interestingly, the frequency of leukemic cells that lack the differentiation markers CD3ε, Ly6C/G, Ter-119 and CD19 (termed L-Lin^−^ cells) was significantly elevated in the spleen when compared to BM or blood (Fig. 1A), suggesting an enrichment of less differentiated leukemic cells in the spleen. Transplantation of total BCR-ABL1-GFP^+^ cells in titrated numbers into lethally irradiated (2 × 6.5 Gy) BL/6 recipients revealed a significant enrichment of leukemia initiating cells in the spleen (Fig. 1B). The frequency of lin^−^, ckit^+^, Sca-1^+^ cells (LSCs) in total L-lin^−^ cells was comparable in spleen and BM (Fig. 1C). However, frequencies of the more primitive LSC subsets LT-LSCs, ST-LSCs and L-MPP1 were increased in the spleen, whereas the frequency of L-MPP2 was not different (Fig. 1D–G, Fig. S2K). To assess the capacity of LSCs to induce CML, we transplanted FACS sorted LSCs from BM and spleen of primary CML mice into sublethally (4.5 Gy) irradiated secondary recipients. All mice receiving spleen LSCs died of CML within 2 months, whereas 50% of recipients of BM LSCs survived long-term (Fig. 1H). This functionally confirms that spleen LSCs harbor a higher frequency of disease initiating stem cells.

The frequency of L-CMPs was increased in the spleen with a concomitant decrease of the more differentiated L-GMPs (Fig. 1I, J, Fig S2L). Taken together, these results demonstrate that the spleen harbors greater numbers of functionally competent, primitive LSCs than the BM with an impaired differentiation of leukemic progenitor cells.

### The microenvironment affects LSPC frequencies

To analyze whether the observed differences in LSPC frequencies are due to cell intrinsic differences of the leukemia cells or due to external cues provided by the microenvironment, we transplanted 5 × 10^4^ primary LSCs from the BM or the spleen into non-irradiated secondary BL/6 recipients. Of note, independent of the LSCs’ origin, an enlarged L-lin- fraction with increased frequencies of LT-and ST-LSCs accumulated in the spleen during the course of the disease (Fig. 1K). Similarly, the differentiation block between L-CMPs and L-GMPs occurred in the spleen independent of the LSCs′ origin (Fig. 1K). These results indicate that the splenic microenvironment differentially affects LSPC accumulation and differentiation compared to the BM microenvironment.

### The spleen contributes to disease progression in the BM

**Fig. 2:**
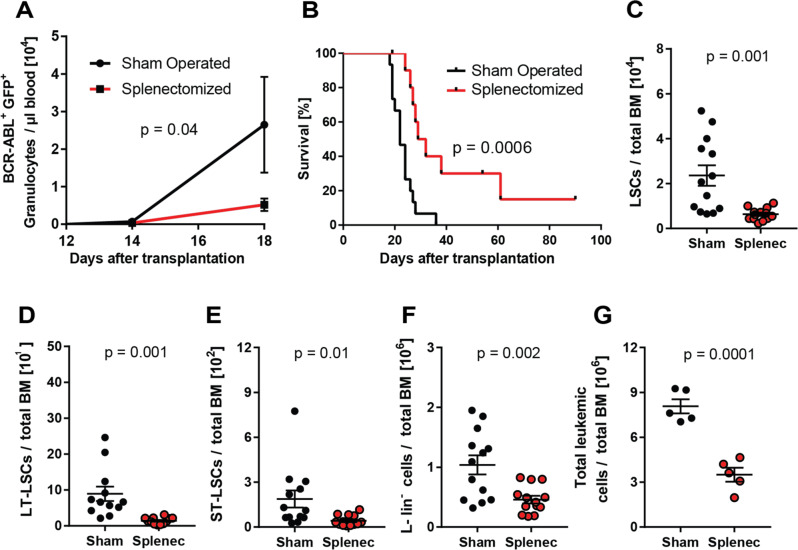
The spleen contributes to disease progression. BL/6 mice were splenectomized or sham-operated 14 days prior to CML induction. **A** Numbers of BCR-ABL1-GFP^+^ granulocytes/µL blood, measured at day 14 and 18 after CML induction. **B** Kaplan–Meier survival curve of primary transplanted, non-irradiated, splenectomized or sham-operated BL/6 recipients. Pooled data from three independent experiments with *n* = 11–15 mice per group are shown. **C**–**G** Absolute numbers of different leukemic cell subsets in the BM of splenectomized and sham-operated animals at day 18 after CML induction. Pooled data from 3 independent experiments with *n* = 13 mice per group are shown. Data are displayed as mean ± SEM. Statistics: 2-way ANOVA (**A**), log-rank test (**B**), unpaired student’s *t* test (**C**–**G**).

To evaluate whether the observed accumulation of disease initiating LSCs in the spleen affects disease development and progression, we induced CML in splenectomized or sham operated BL/6 mice. Fourteen days after transplantation, splenectomized and sham-operated animals showed similar numbers of leukemic granulocytes in blood, suggesting a comparable engraftment of LSCs (Fig. 2A, 370 ± 260/μl in sham operated vs. 364 ± 260/μl in splenectomized animals, p = n.s.). However, CML progressed significantly slower in splenectomized mice, resulting in lower BCR-ABL1-GFP^+^ granulocyte counts in blood 18 days after transplantation (Fig. 2A). Splenectomized animals had a significant survival advantage, with 20% surviving more than 90 days, whereas sham-operated animals all died of leukemia within 40 days (Fig. 2B). Next, we analyzed the LSPC compartment in the BM of splenectomized and sham-operated animals 18 days after CML induction. Interestingly, all LSPC subsets as well as total BM cellularity were significantly reduced in splenectomized CML mice (Fig. 2C–G and Fig. S3A–E). These findings demonstrate that the spleen contributes to disease progression by expanding the pool of LSCs.

### Transcriptomic analysis of LSPCs in the spleen reveals enrichment in stemness and decreased lineage commitment

**Fig. 3:**
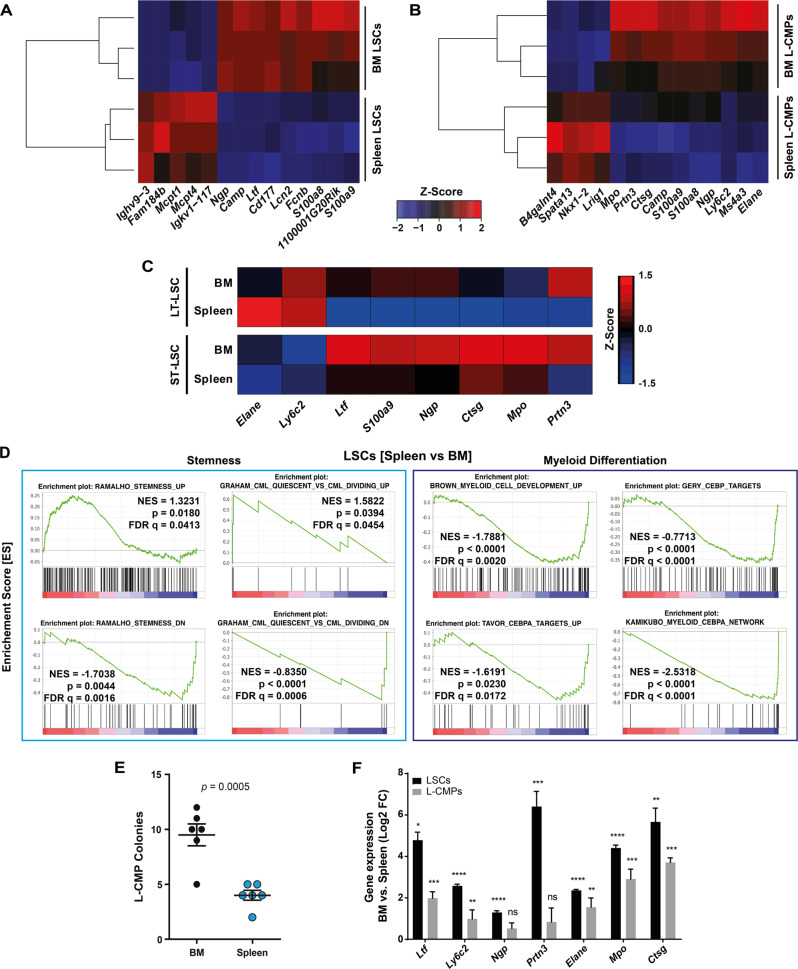
Transcriptomic analysis of LSPCs in the spleen reveals enrichment in stemness and decreased lineage commitment. **A**, **B** Heatmap visualizing the expression of the significantly differentially expressed genes in LSCs and L-CMPs. Rows and columns were grouped using unsupervised hierarchical clustering. **C** Expression of genes involved in myeloid expression in LT- and ST-LSCs from BM and spleen. Data were clustered using standard Euclidean’s method based on the average linkage and heatmaps were generated according to the standard normal distribution of the values. **D** Gene set enrichment analysis (GSEA) representing the normalized enrichment score (NES) and false discovery rate score (FDR) of gene sets linked to stemness and myeloid differentiation for LSCs (spleen vs. BM). **E** 10^3^ FACS-purified L-CMPs from spleen and BM of CML mice were plated into methylcellulose and colonies were counted 7 days later. One representative experiment out of 3 is shown with *n* = 6 mice per group. **F** LSCs and L-CMPs from spleen were co-cultured for 36 h with spleen and BM flush as supernatants. Depicted are log_2_ fold changes in gene expression in LSCs and L-CMP cultured with BM or spleen flush supernatant. One representative experiment out of 2 is shown with *n* = 3 biological replicates. Data are displayed as mean ± SEM. Statistics: Unpaired student’s *t* test (**E**, **F**).

Next, we compared gene expression of FACS-purified LSCs and L-CMPs in the spleen and BM 18 days after CML induction using RNA-Seq. Fourteen genes were differentially expressed between BM and spleen in both LSCs and L-CMPs (Fig. 3A, B). Interestingly, *Ltf* and *Ngp*, which are induced upon myeloid differentiation [23, 24], and *S100a9*, which induces differentiation of acute myeloid leukemia cells [25] were downregulated in LSCs isolated from the spleen compared to those from BM (Fig. 3A). Similarly, *Ltf*, *Ngp, S100a9*, as well as the myeloid differentiation markers *Mpo, Elane, Ctsg, Prtn3* and *Ly6c2* were downregulated in splenic L-CMPs (Fig. 3B) [23, 24]. In addition, the differentially expressed genes that are related to myeloid differentiation were analyzed in LT- and ST-LSCs from spleen and BM using quantitative real time PCR. Most of the analyzed genes involved in myeloid differentiation were downregulated in stem cells from the spleen when compared to the BM (Fig. 3C), indicating that LT- and ST-LSCs are functionally different in spleen and BM and that the observed differences in gene expression of total LSCs (Fig. 3A) do not only reflect a numerical increase of LT-LSCs in the spleen (Fig. 1D, E). To analyze the functional role of *S100a9* and *Ltf* in myeloid differentiation, we performed shRNA knockdown experiments (Fig S4A) in total LSCs and assessed colony formation capacity and CD11b expression. Knockdown of *S100a9* and *Ltf* increased the self-renewal capacity of LSCs leading to elevated numbers of colonies and decreased expression of the differentiation marker CD11b, indicating that *S100a9* and *Ltf* induce differentiation of CML LSCs (Fig. S4B, C)

Geneset enrichment analysis (GSEA) revealed a significant up-regulation of genes involved in stem cell maintenance [26, 27] in Spleen-LSCs versus BM-LSCs (Fig. 3D). Furthermore this was accompanied by a down-regulation of genes involved in myeloid differentiation, especially genes induced by C/EBP transcription factors (Fig. 3D) [28–30]. Together, this further supports our findings that spleen LSCs are more primitive than BM LSCs. GSEA also revealed a downregulation of genes involved in myeloid differentiation in splenic L-CMPs (Fig. S4D) [29]. These data are consistent with a block of differentiation in splenic L-CMPs. To support these results, we isolated BM and spleen L-CMPs from CML mice and assessed their differentiation potential in vitro in colony forming assays. L-CMPs from the spleen formed significantly lower numbers of colonies compared to BM L-CMPs, indicating impaired differentiation of splenic L-CMPs (Fig. 3E).

In addition, we flushed CML BM and spleen and used the cell-deprived flow-through as a culture medium for L-CMPs and LSCs. After 36 h of culture, expression of myeloid differentiation genes was measured. Interestingly, L-CMPs and LSCs cultured in the presence of BM flush showed a consistent upregulation of *Ltf, Ly6c2, Ngp, Prtn3, Elane, Mpo* and *Ctsg* compared to L-CMPs cultured with spleen flush (Fig. 3F).

To further characterize the leukemic compartment in BM and spleen, we quantified cytokines, chemokines and growth factors in cell suspensions of CML BM and spleen supernatants using ELISA and Multiplexing LASER Bead Assay. In order to compare cytokine concentrations between BM and spleen, the volume to flush the organs was adjusted based on cell numbers to obtain equal concentrations of cells/ml. Although this method adjusts for different cell numbers, it does not reflect physiological in vivo concentrations of cytokines. A total of 14 cytokines could neither be detected in the BM nor in the spleen. 23 cytokines were upregulated in spleen and 7 were upregulated in BM cell suspensions (Fig. S5). Cytokines that promote myeloid differentiation, mainly IL-3 and IL-6 [31–33], were upregulated in the BM and several cytokines known to be implicated in inflammation, tumorigenesis, and HSCs and LSCs functions, such as IL-10, CCL2, CCL3, CCL5, TNF- α, Flt3L and SCF were upregulated in the spleen (Fig. S5) [34–40].

Taken together, our results suggest that the spleen provides a microenvironment that confers stemness and lacks differentiation signals for LSPCs.

### Spleen LSCs are quiescent and resistant to TKI therapy

**Fig. 4:**
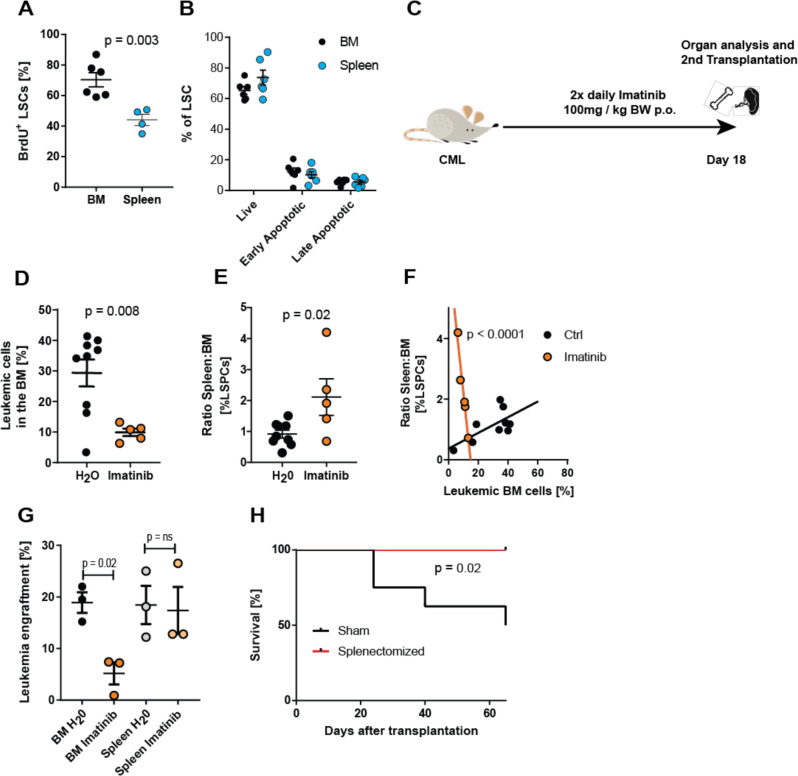
LSCs in the spleen are more quiescent than in the BM and resistant to TKI therapy. **A** BrdU incorporation of LSCs in the spleen and BM 18 days after CML induction and 12 h after intraperitoneal BrdU administration. One representative of 4 independent experiments with *n* = 4–6 mice per group is shown. **B** Frequency of viable and apoptotic LSCs in the BM and spleen of CML mice 18 days after CML induction. Viable cells are defined as Annexin V^−^, 7-AAD^−^, early apoptotic cells as Annexin V ^+^, 7-AAD^−^, and late apoptotic cells as Annexin V ^+^, 7-AAD^+^. One representative out of two independent experiments with *n* = 5 mice per group is shown. **C** Experimental model. BL/6 CML mice were treated with imatinib (100 mg/kg BW) or vehicle (H_2_O) twice daily by oral gavage starting 4 days after CML induction. **D** Degree of BM infiltration with leukemic cells at day 18 after CML induction. **E** To compare resistance between BM and spleen, the spleen:BM-ratio of LSPCs was calculated. **F** Relationship between leukemia load in the BM and the spleen:BM-ratio of LSPCs. Straight lines represent linear regression calculations, dashed lines represent upper and lower 95% confidence intervals. Pooled data from two independent experiments with *n* = 5–8 mice per group are shown. **G** Leukemia load at day 25 in peripheral blood of lethally irradiated secondary recipient mice that received 2 × 10^6^ whole-BM or -spleen cells from imatinib- or vehicle-treated primary CML mice (*n* = 3). **H** Kaplan–Meier survival curve of imatinib-treated CML mice that were splenectomized or sham-operated prior to CML induction. *n* = 8 mice per group. If not otherwise stated, data are displayed as mean ± SEM. Statistics: unpaired Student’s *t* test, (**A**, **D**, **E**), one-way ANOVA (**B**, **G**), linear regression (**F**), and log-rank test (**H**).

Quiescent and more primitive LSCs are resistant to TKI therapy [41–43]. It is well documented that the BM microenvironment contributes to the quiescence of LSCs and therefore to therapy resistance [3, 4]. Since spleen LSCs had a stronger stemness gene expression signature and contained a higher frequency of LT-LSCs, we reasoned that the spleen might contribute to CML therapy resistance. We therefore first analyzed the proliferation of LSCs in spleen and BM 18 days after CML induction by assessing BrdU incorporation. Interestingly, fewer spleen LSCs incorporated BrdU than BM LSCs (Fig. 4A). In contrast, the rate of apoptosis was comparable between BM and spleen LSCs as assessed by Annexin V and 7-AAD stainings (Fig. 4B).

Next, we analyzed the effectiveness of imatinib treatment on BM and spleen LSPCs. BL/6 CML mice were treated with imatinib or water for 14 days starting at day 4 after transplantation (Fig. 4C). As expected, BM and spleen infiltration by BCR-ABL1-GFP^+^ leukemic cells was reduced after imatinib treatment and absolute LSPC numbers were reduced in BM and spleen (Fig. 4D, Fig S6A–E). More interestingly, LSPC frequencies in the BM were significantly lower than in the spleen after imatinib treatment, as indicated by an increasing ratio of spleen:BM LSPCs (Fig. 4 E, Fig S6F, G). Consistently, the ratio spleen:BM LSPCs increased with the effectiveness of the therapy (Fig. 4F). Taken together, this indicates that spleen LSPCs are more resistant to TKI treatment. To functionally prove the difference in resistance between spleen and BM LSPCs, we secondarily transplanted 2 × 10^6^ total BM or spleen cells from imatinib or water treated CML mice into lethally irradiated BL/6 recipients. Engraftment of CML was assessed 25 days after secondary transplantation and revealed comparable leukemia engraftment in mice receiving BM or spleen cells from water treated mice (Fig. 4G). Imatinib treatment of primary CML mice reduced the engraftment of transplanted BM cells but not spleen cells in secondary recipients. This indicates that imatinib treatment did not significantly reduce the number of disease-initiating cells in the spleen (Fig. 4G).

Fifty percent of CML patients who discontinue TKI treatment relapse due to resistant LSCs [44]. Since our experiments revealed that most of the resistant LSCs are located in the spleen, we assessed the impact of splenectomy in combination with imatinib treatment on disease outcome. None of the splenectomized animals died during the observed period, while 50% of sham operated animals died due to CML progression (Fig. 4H). Taken together, our results demonstrate that the microenvironment in the spleen renders LSCs quiescent and resistant to TKI therapy.

### Red pulp macrophages constitute the spleen niche and induce LSC quiescence and resistance to treatment with TKI

**Fig. 5:**
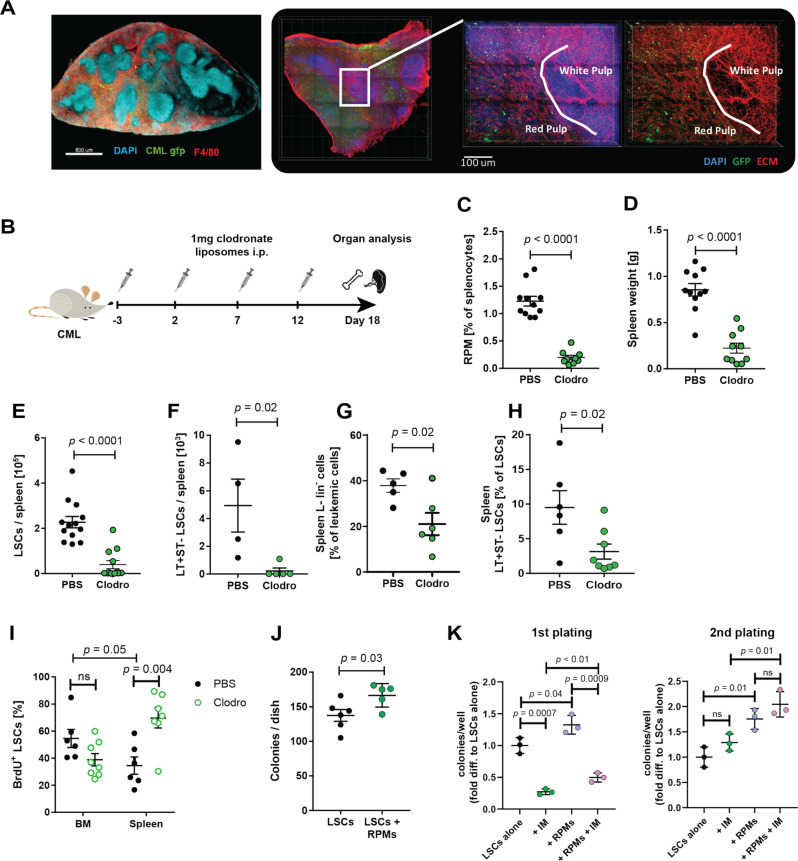
LSCs in the spleen are dependent on macrophages and located in the red pulp. **A** 3D confocal laser scanning microscopy of the spleen 10 days after CML induction. Representative images from 3 independent experiments with *n* = 6 mice are shown. **B** Experimental model. BL/6 CML mice were treated with clodronate liposomes (1 mg) or vehicle (PBS) every 5th day by intraperitoneal injection starting 3 days prior to CML induction. Spleen and BM were analyzed 18 days after CML induction. **C** Frequency of RPMs after clodronate treatment. **D**–**H** Spleen weight, numbers and frequencies of LSCs, LT-, ST-LSCs and L-lin^−^ cells. Pooled data from 2 to 3 independent experiments with *n* = 5–13 mice are shown. **I** Incorporation of BrdU in LSCs of the BM and spleen of clodronate or PBS treated CML mice (*n* = 6–8 mice). **J** In total, 1.5 × 10^3^ FACS-purified LSCs were co-incubated with RPMs for 48 h and subsequently plated into methylcellulose. Numbers of colonies were counted after 7 days. Pooled data of two independent experiments with *n* = 5–6 samples per group are shown. **K** For 1st platings, 1.5 × 10^3^ FACS-purified LSCs were co-incubated overnight with 1 × 10^4^ FACS-purified RPMs in the presence or absence of imatinib and plated into methylcellulose. Colonies were counted after 7 days. For 2nd platings, colonies were washed and 1 × 10^4^ cells were plated into methylcellulose. Colonies were counted after 7 days. One representative experiment with *n* = 3 biological replicates is shown. Data are displayed as mean ± SEM. Statistics: Unpaired student’s *t* test.

Next, to characterize the leukemic microenvironment in the spleen in more detail, we performed 3D confocal laser scanning microscopy 10 days after CML induction. Interestingly, BCR-ABL1-GFP^+^ leukemia cells were exclusively located in the red pulp of the spleen (Fig. 5A). Red pulp macrophages (RPMs) were recently described to be important for HSC localization and regulation during extramedullary hematopoiesis in the spleen [13, 45]. To investigate the effect of RPMs on LSPCs, we depleted macrophages in CML mice using clodronate liposomes (Fig. 5B) [46]. Eighteen days after CML induction and after 4 injections of clodronate, RPMs (F4/80^+^, CD11b^int^) were substantially reduced (Fig. 5C). Depletion of macrophages significantly reduced spleen weights as an indicator of splenic leukemia burden (Fig. 5D). Total numbers and frequencies of LSCs, LT- and ST-LSCs, and L-lin^−^ cells were significantly reduced in the spleen after treatment with clodronate (Fig. 5E–H)

Since we showed that the splenic microenvironment induced quiescence of LSCs, we analyzed BrdU incorporation in LSCs of the spleen and BM after macrophage depletion. Interestingly, clodronate-induced macrophage depletion increased the proliferation of LSCs in the spleen, whereas LSCs in the BM were not affected (Fig. 5I). Next, we analyzed the impact of RPMs on LSCs in vitro. Pre-incubation with RPMs overnight significantly increased the colony formation capacity of LSCs (Fig. 5J) and resulted in a decreased susceptibility of LSCs against TKI treatment with imatinib (Fig. 5K).

### Depletion of RPMs reduces spleen LSCs

**Fig. 6:**
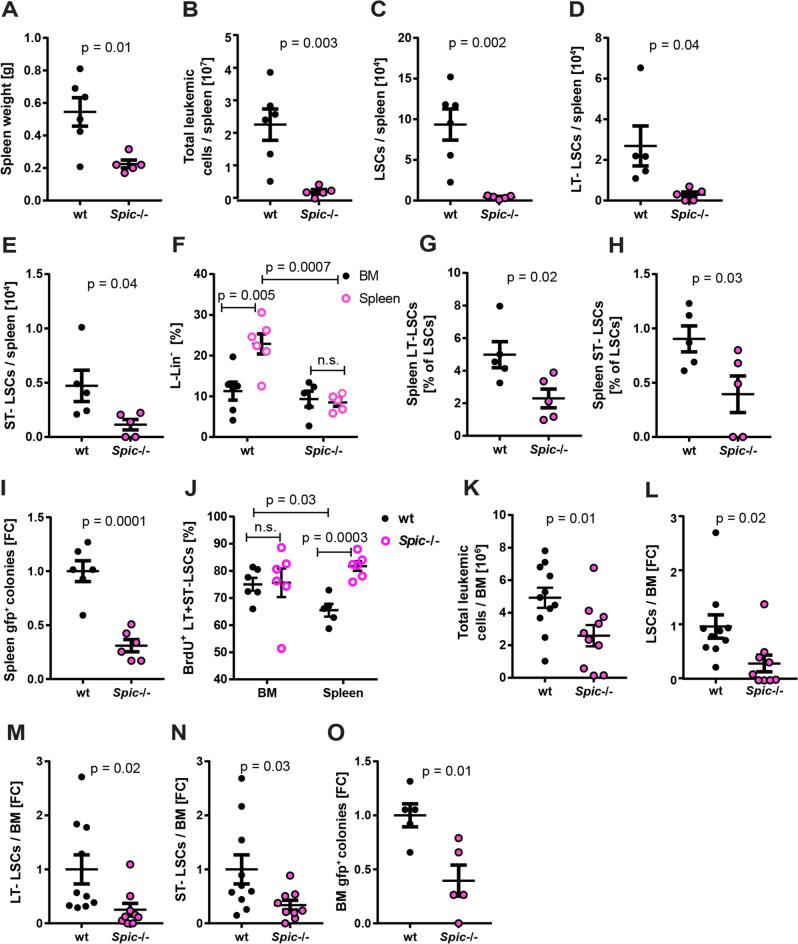
Red pulp macrophages retain LSCs in the spleen. *Spic*^wt/wt^ donor LSK cells were transduced with BCR-ABL1-GFP and transplanted into *Spic*^wt/wt^ and *Spic*^–/–^ mice. The leukemic compartment of the CML mice was analyzed 15 days after CML induction. **A** Spleen weight. Absolute numbers of (**B**) total leukemic cells, (**C**) LSCs, (**D**) LT-LSCs, (**E**) ST-LSCs in spleen. One representative experiment out of 3 is shown with *n* = 5–6 mice per group. **F** Frequency of L-lin^−^ cells in the spleen and BM. **G**, **H** Frequency of spleen LT- and ST-LSCs. One representative experiment out of 3 is shown with *n* = 5–6 mice per group. **I** 10^5^ total spleen cells from CML mice were plated into methylcellulose and BCR-ABL1-GFP^+^ colonies were enumerated after 7 days. Fold change (FC) numbers of colonies from *Spic*^wt/wt^ and *Spic*^−/−^ spleen cells are shown. One representative experiment out of 2 is shown with *n* = 6 mice per group. **J** Incorporation of BrdU in LSCs of the BM and spleen of *Spic*^wt/wt^ and *Spic*^–/–^ CML mice. One experiment with *n* = 8 mice per group is shown. **K** Total leukemic cells in the BM of *Spic*^wt/wt^ and *Spic*^–/–^ CML mice. Pooled data from 2 independent experiments with *n* = 10–11 mice per group is shown. FC of cell numbers of **L** BM LSCs, **M** LT-LSCs and **N** ST-LSCs. **O** 10^5^ total BM cells from CML mice were plated into methylcellulose and BCR-ABL1-GFP^+^ colonies were enumerated after 7 days. FC of numbers of colonies from *Spic*^wt/wt^ and *Spic*^–/–^ BM cells are shown. One representative experiment out of 2 is shown with *n* = 5 mice per group. Data are displayed as mean ± SEM. Statistics: Unpaired student’s *t* test.

To assess the impact of RPMs on LSCs in the spleen more specifically, we made use of the Spi-C loss of function mouse (*Spic*^–^), which lacks RPMs [15]. BCR-ABL1-GFP transduced *Spic*^wt/wt^ LSKs were transplanted into *Spic*^−/−^ and *Spic*^wt/wt^ recipient mice and CML development in spleen and BM was analyzed. The lack of RPMs resulted in a decreased leukemia load in the spleen, as indicated by lower spleen weights and decreased numbers of total leukemic cells (Fig. 6A, B). More importantly, total numbers of spleen LSCs, LT- and ST-LSCs, as well as frequencies of L-lin^−^, LT- and ST-LSCs were significantly reduced in *Spic*^−/−^ CML mice (Fig. 6C–H). To further confirm these findings, we assessed the leukemic colony formation capacity of total splenocytes from *Spic*^−/−^ and *Spic*^wt/wt^ CML mice. Spleen cells from *Spic*^−/−^ mice formed significantly fewer BCR-ABL1-GFP^+^ colonies compared to *Spic*^wt/wt^ cells (Fig. 6I). Similar to clodronate-induced macrophage depletion, the specific absence of RPMs in *Spic*^*−/−*^ mice increased proliferation of LSCs in spleen but not BM (Fig. 6J). As shown in Fig. 2, splenectomy reduced the number of LSCs and leukemia burden in the BM. Similarly, depletion of RPMs substantially reduced leukemia burden, numbers of LSCs as well as colony formation capacity in the BM (Fig. 6K–O). It was shown that macrophages retain HSC in the spleen via VCAM-1/VCA4 signaling [13]. However, treatment of CML mice with a monoclonal VCAM-1 blocking antibody did not reduce leukemia burden or numbers of LSCs in the spleen (Fig. S7A–C).

To characterize RPMs in more detail, high-dimensional single cell mass cytometry of naïve and CML RPMs was performed. This analysis revealed a downregulation of colony stimulating factor 1 receptor (CSF1R, CD115) in CML RPMs as only consistent phenotypical difference (Fig S8A, B). However, CML RPMs produced different cytokines and chemokines known to be associated with LSC and HSC maintenance, such as IL10, CCL3, CCL4, CCL5, CXCL1, TNF-α or SCF (Fig. S8C).

Taken together, our results suggest that leukemic RPMs form a niche that retains LSCs in the spleen, contributes to their maintenance and quiescence as well as to disease progression and resistance to therapy.

### LSCs co-localize with macrophages in human CML spleens

**Fig. 7:**
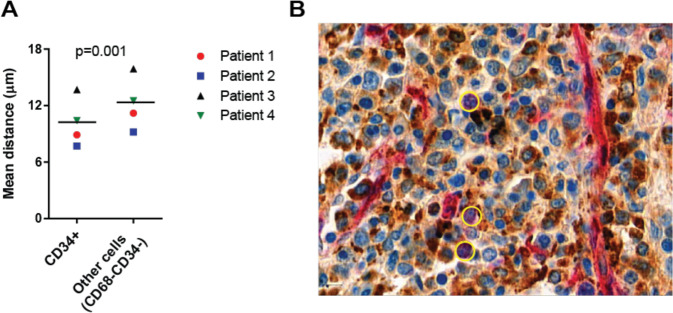
LSCs co-localize with macrophages in human CML spleens. **A** Mean distance between CD68^+^ macrophages and CD34^+^ human stem/progenitor cells or CD68^−^CD34^−^ cells in 4 CML patients. **B** Representative immunohistochemical staining (patient 1) for CD68 (brown) and CD34 (red). Stem/progenitor cells (CD34^+^ non-vascular cells with a nuclear morphology compatible with immature cells (large nuclei, open chromatin)) are annotated with yellow circles. Data are displayed as mean ± SEM. Statistics: Paired student’s *t* test.

To extend our findings to human CML, we studied the localization of CD34^+^ stem/progenitor cells and CD68^+^ macrophages in the spleen of 4 CML patients who underwent splenectomy (Table S2). All spleens analyzed showed massive infiltration of the red pulp by myeloid cells. Immunohistochemistry followed by cell segmentation and proximity analysis revealed that CD34^+^ stem/progenitor cells were located closer to CD68^+^ macrophages than to all other CD68^-^ cells (Fig. 7A–B). These data indicate that macrophages are part of the LSC niche in the spleen of CML patients.

## Discussion

Specialized niches in the BM regulate HSC quiescence, self-renewal and differentiation [5, 47]. There is increasing evidence that LSCs hijack HSC niches as they depend on similar extrinsic signals for their maintenance [4, 48]. Splenomegaly is a hallmark of CML that results from the extensive infiltration with leukemic cells and EMH and has been identified as a negative prognostic marker [49]. Whether splenectomy in CML patients results in an improved disease control is controversial. While small retrospective studies suggested a survival advantage [50, 51], a prospective randomized study in early phase CML did not reveal a survival benefit [52].

However, the contribution of the spleen in the pathogenesis of CML is poorly understood. Compared to healthy BM, CXCL12 levels on mesenchymal stem cells in the BM microenvironment in CML are decreased, resulting in a reduced LSC-retention in the BM and egress to the circulation [22, 53]. We now document that LSCs in the spleen reside in a specialized niche that is formed by RPMs. RPMs lead to an accumulation of large numbers of quiescent LSCs that contribute to the progression of the disease and resistance to the therapy with TKI. Importantly, since irradiation has been shown to alter the microenvironment and the production of inflammatory cytokines [54, 55], recipient mice were not irradiated in our study. Lethal irradiation reduces the majority of the immune cells in the spleen and BM, leads to a loss of endothelial cells, vessel dilatation, cytokine release and even bone loss [56, 57]. This may at least partially explain the discrepant findings in other CML models using lethal irradiation. Zang et al., which used a genetic-driven CML model, documented an accumulation of LSCs in the spleen but a similar or lower frequency of LT-LSCs [22]. Moreover, Agarwall et al found an increased resistance of BM LT-LSCs upon nilotinib treatment that depended on CXCL12 release from MSCs, whereas splenic CML cells were more susceptible to TKI treatment [53]. In contrast, similar to our results, Schemionek et al. documented an increased resistance of splenic leukemic cells compared with BM leukemic cells towards imatinib [14]. This may reflect the previously documented differences of susceptibility of LSCs towards the various TKIs [58]. Thus, the kinetics of the CML development, the irradiation of the recipient and the TKI studied may influence the number and function of LSCs in the spleen.

Tumor-associated macrophages (TAM) are increasingly recognized to promote tumor progression in solid tumors and hematologic malignancies via multiple mechanisms, such as increased proliferation and decreased apoptosis of tumor bulk cells or by inducing angiogenesis and immunosuppression [59, 60]. However, little is known about the impact of TAM on LSCs. In this work, we found RPMs to be a crucial component of the LSC-niche in the spleen. Similar as published for HSCs [13], LSCs were exclusively located in the red pulp of the spleen and retained by RPMs. However, the precise mechanism of LSC-retention by RPMs remains unknown, a VCAM-1 dependent retention as shown by Dutta et al. for HSCs [13] could not be seen in our study for LSCs. Remarkably, RPM induced quiescence of splenic LSCs and thereby contributed to therapy resistance [41]. In RPM-deficient *Spic*^*−/−*^ mice, total leukemia burden and numbers of LSCs were also decreased in BM, similar to splenectomized CML mice, suggesting a back-and-forth trafficking between spleen and BM. The accumulation and retention of primitive leukemia cells in the spleen contributes to disease progression. Indeed, we found several chemokines previously described to promote LSC growth in CML-BM, such as CCL-3, CCL-4, and TNF-α to be expressed in the splenic microenvironment and produced by RPMs in CML. In addition, IL1-α, Il-12 and VEGF, that have been shown to support the growth of murine erythroleukemia cells in the spleen, were also upregulated in the spleen in our study [61]. The identification of the cytokines produced by RPMs that induce quiescence in LSCs requires further studies.

Mass cytometry analysis revealed downregulation of CD115/CSFR1 in CML RPMs This is rather surprising since TAM of the M1 type, which are pro-inflammatory and activate the immune system towards a tumoricidal state, are known to be CD115 dependent, whereas M2 differentiated TAMs rely on CD115, suppress immune effector functions and promote tumorigenesis. Previous work showed that blocking the CSFR1–CSF-1 axis of TAM decreased tumor load in hematological malignancies and is an attractive clinical target [62–64]. Our results now show that CD115-independent RPMs in CML mice contribute to disease by producing cytokines that expand LSCs. Similarly, cytotoxic T lymphocytes have paradoxical effects on CML LSCs and promote LSC expansion by the production of effector cytokines such as IFNγ [4, 65].

Gene expression analysis revealed a downregulation of several genes involved in myeloid differentiation and enrichment of stemness genes in splenic L-CMPs and LSCs, respectively. As a consequence, myeloid differentiation of leukemic progenitor cells was reduced in the spleen, leading to an accumulation of leukemia initiating LSCs and a reduction of more differentiated progenitors. We similarly documented a reduced expression of genes involved in myeloid differentiation in LT-LSCs and ST-LSCs in the spleen versus BM, suggesting that the spleen microenvironment directly regulates the function of LT-LSCs. However, due to the limited numbers in BM LT-LSCs we could not experimentally validate a different function in limiting dilution transplantation experiments to secondary recipients. Recently, Rowe et al. demonstrated that hematopoiesis and lineage commitment are influenced by the local microenvironment [24]. During embryonic development, MEPs accumulated in the fetal liver, whereas fetal liver GMPs were reduced compared to the BM. In addition, and similar to what we have found for leukemic differentiation in the spleen, gene expression analysis revealed upregulation of myeloid lineage–specific transcripts in CMPs from the BM compared to the fetal liver.

It is well known that the BM microenvironment contributes to resistance against conventional therapies. For example, CXCL12 expressed by niche cells in the BM attracts AML and CML cells, promotes survival and quiescence and resistance to therapy [66, 67]. E-selectins on the BM endothelium support homing of CML LSC via CD44 and lead to decreased proliferation of leukemia cells and reduced susceptibility to TKI treatment [68]. Furthermore, BM mesenchymal stem cells were shown to protect CML LSPC from TKI treatment by inhibiting apoptosis and maintaining colony-forming ability [69]. Our study now indicates that RPMs in the spleen crucially contribute to the resistance of CML LSCs to TKI by maintaining LSC quiescence.

## Supplementary information

Suppl. Material
