# Supplementary material for: Splenic red pulp macrophages provide a niche for CML stem cells and induce therapy resistance
Source: Leukemia. 2022 Sep 26;36(11):2634–46. doi: 10.1038/s41375-022-01682-2 (PMC7613762; doi:10.1038/s41375-022-01682-2)
Supplement: Supplementary file 1 — Suppl. Material [file 41375_2022_1682_MOESM1_ESM.pdf]

Supplementary Material

Supplementary Methods

Antibodies for flow cytometry

αckit-APC-Cy7 (cat. 105826, clone 2B8, 1:300), and -PE-Cy7 (cat. 105813, clone 2B8, 1:600) αSca-1-PerCp-Cy5.5 (cat. 108123, clone D7, 1:600), αCD16/32-PE-Cy7 (cat. 101318, clone 93, 1:200), , αCD135-Alexa Fluor 700 (cat. 135306, clone A2F10, 1:100), αCD48-APC (cat. 103412, clone HM48-1, 1:100), CD150-PE (cat. 115904, clone TC15-12.2, 1:100), αF4/80-APC (cat. 123116, clone BM8, 1:200), αCD19-APC-Cy7 (cat. 115530, clone 6D5, 1:300) and -biotin (cat. 115503, clone 6D5, 1:300), αCD3ε-biotin (cat. 100304, clone 145-2C11, 1:300), αLy6G/C-PE (cat. 108407, clone RB6-8C5, 1:400) and -biotin (cat. 108404, clone RB6-8C5, 1:300), and αTer-119-biotin (cat. 116203, clone Ter-119, 1:300) were from BioLegend. αCD34-eFluor 450 (cat. 48-0341-82, clone RAM34, 1:100) was from Thermo Fisher. αCD11b-PE (cat. 12-0112-82, clone M1/70, 1:300) was from eBioscience and αStreptavidin-BD Horizon V500 (cat. 561419, 1:1000) was from BD Pharmingen.

Laser scanning microscopy

Mouse spleens were isolated, cleaned and immersed in PBS/2% paraformaldehyde for 6h at 4°C, followed by a dehydration step in 30% sucrose for 72 h at 4°C. Spleens were then embedded in cryopreserving medium and snap frozen in liquid nitrogen. Spleen specimens were iteratively sectioned using a cryostat until fully exposed. The OCT block containing the spleen was then reversed and the procedure was repeated on the opposite side until fully exposed. Once spleen slices were generated, the remaining OCT medium was removed by incubation and washing of the spleen slices in PBS 3 times for 5 minutes. Prior to immunostaining, slices were incubated in blocking solution (0.2% Triton X-100, 1% bovine serum albumin, 10 % donkey serum, in PBS) overnight at 4°C. Primary antibody stainings were performed in blocking solution for 3 days at 4°C, followed by overnight washing in PBS. Secondary antibody stainings were performed for another 3 days at 4°C in blocking solution but in the absence of BSA to avoid cross-absorption.

Immunostained splenic slices were successively washed in PBS overnight and incubated in RapiClear 1.52 for a minimum of 6h. Spleen slices were mounted on glass slides using vacuum grease. Confocal microscopy was performed with 10x (HCX PL FLUOTAR), 20x (HC PL APO CS2) and 63x (HCX PL APO CS2) using SP5 and SP8 Leica confocal microscopes equipped with hybrid detectors.

Immunohistochemistry

Formalin-fixed, paraffin-embedded (FFPE) spleen samples from four CML patients undergoing splenectomy between 1990 and 2002 were identified in the archive of the Institute of Pathology, University of Bern, Switzerland (Table S2). From each patient, one representative block was taken for hematoxylin and eosin staining and immunohistochemistry (IHC). IHC stainings were performed on a Leica BOND RX automated immunostainer according to the manufacturer's instructions (Leica Biosystems). Thin sections (1-2um) of FFPE tissue were pre-treated by boiling at 100°C in citrate buffer, pH 6.0 for 30 min. Double stainings for CD68 and CD34 were performed sequentially. First, slides were incubated with rabbit anti-human CD68 (clone KP1, Dako) at a dilution of 1:5000 in BOND primary antibody diluent (Leica Biosystems), followed by visualization using the BOND Polymer Refine DAB Detection kit (DS9800, Leica Biosystems). Then, slides were counterstained with mouse anti-human CD34 (clone QBEnd/10, Cell Marque) at a dilution of 1:200, followed by visualization using the BOND Polymer Refine Red Detection kit (DS9390, Leica Biosystems). Finally, nuclei were counterstained with hematoxylin.

Slide digitization, cell annotation and proximity analysis

Slides were scanned using an Aperio Scanscope CS digital slide scanner (Leica Biosystems) and analyzed using QuPath software (version 0.1.2). For each spleen sample, an 8 mm^2 area was analyzed. For cell segmentation, detection of $CD68^+$ and $CD68^-$ cells (including the $CD34^+$) and identification of centroids, the QuPath positive cell detection algorithm was used. The quality of segmentation and positive and negative cell detection was analyzed and confirmed for each case. $CD34^+$ non-vascular cells with a nuclear morphology compatible with immature cells (large nuclei, open chromatin) – "$CD34^+$ LSCs" – were identified and annotated by hand by a board-certified surgical pathologist

(C.M.S.). Proximity analysis was performed by a mathematician (S.S.B.) using Python's SciPy package (Python Software Foundation). For each CD68-cell, the minimum Euclidean distance (in two dimensions) between its centroid and the centroids of all the CD68+ cells was computed. The centroids of manually annotated $CD34^{+}$ stem/progenitor cells were identified in the $CD68^{-}$ cells by alignment of centroids and these cells were removed from the $CD68^{-}$ cells.

Transcriptome analysis using next generation RNA sequencing (RNA-Seq)

Total RNA was extracted from FACS purified LSCs and L-CMPs isolated from the BM and the spleen (total of 12 samples) using the RNeasy Micro Kit (QIAGEN AG, Switzerland). Total RNA was quality-checked using the Bioanalyzer instrument (Agilent Technologies, Santa Clara, CA, USA) using the RNA 6000 Pico Chip (Agilent, cat. 5067-1513) and quantified by Fluorometry using the Quantus Fluorometer (Promega, Madison, WI, USA) with the kit Quantifluor RNA System (cat. E3311, Promega). Library preparation was performed from total RNA using the SMART-Seq v4 Ultra Low Input RNA Kit for Sequencing (cat. 634891, Takara Bio). Libraries were quality-checked on the Fragment Analyzer (Advanced Analytical, Ames, IA, USA) using the High Sensitivity NGS Fragment Analysis Kit (cat.DNF-474, Advanced Analytical). Samples were pooled to equal molarity and the pool was quantified by fluorometry, to be loaded at a final concentration of 2 pM on the NextSeq 500 instrument (Illumina). Samples were sequenced using the NextSeq 500 High Output Kit 75-cycles (Illumina, cat. FC-404- 1005) and primary data analysis was performed with the Illumina RTA version 2.4.11 and bcl2fastq v2.20.0.422.

The RNA-seq data was assembled by SeqMan NGen software v.15. Data normalization and differential gene expression analysis was performed in R using the EdgeR package. The LSC and CMP datasets were analyzed independently. First, read counts were normalized to counts per million (CPM). Next, a tagwise dispersion was calculated. Finally, differential gene expression was analyzed using quasi-likelihood F-tests and likelihood ratio tests. Genes with significant difference in their expression at FDR-$p<0.05$ and fold change differences of >1.5 were considered significantly changed.

VCAM blockade

Mice were treated i.v. with 10mg/kg VCAM-1 antibody (Bio X Cell , cat. BE0027, clone M/K 2.7) or IgG control (Sigma) on day 0, 5, 10 and 15 after CML induction.

Cytokine quantification

Cytokines, chemokines and growth factors were measured in cell-deprived flow-through of CML BM or spleen and in supernatants of *in vitro* cultured RPMs. In detail, long bones were flushed with 0.5 ml DMEM media. Spleens were smashed through cell strainers and the volume of DMEM was adjusted based on cell numbers to obtain equal concentrations (cells/volume) as in BM samples. The suspensions were centrifuged and the supernatants used for measurements or cell culture (see below). $5x10^4$ RPMs from CML mice were cultured in DMEM supplemented with 10% FCS and 1% Glutamine and Pen/Strept for 36 hours and supernatants were used for measurements. Eotaxin, Erythropoietin, , Fractalkine, G-CSF, GM-CSF, IFNB1, IFNγ, IL-1α, IL-1β, IL-2, IL-3, IL-4, IL-5, IL-6, IL-7, IL-9, IL-10, IL-11, IL-12 (p40), IL-12 (p70), IL-13, IL-15, IL-16, IL-17, IL-20, CXCL10, CXCL1, CXCL2, CXCL5, CXCL9, CCL2, CCL3, CCL4, CCL5, CCL12, CCL17, CCL19, CCL20, CCL 21, CCL22, , M-CSF, LIF, TIMP-1, TNFα, and VEGF were measured using the Multiplexing LASER Bead Assay (Eve Technologies). ELISA assays were performed for SFC (Ray Biotech, Norcross, GA, cat. ELM-SCF) and Flt-3L (Ray Biotech, cat., ELM-Flt3L) according to manufacturer's protocol. Concentrations were calculated using standard curves.

Culture of LSCs/CMPs with supernatant

$3x10^3$ LSCs or L-CMPs from the spleen were plated in 96-well plates and 200 ul of BM or spleen flow-through (generated as described above) was used as a growth medium. Gene expression was measured after 36 hours by qPCR.

Colony assays

FACS-purified GFP^+ LSCs or CMPs ($1.5x10^3$ each) were plated into methylcellulose (cat. 03134, STEMCELL Technologies) supplemented with 15% FCS, 20% BIT (50 mg/ml BSA in IMDM, 1.44

U/ml rh-insulin (Actrapid; Novo Nordisk), and 250 ng/ml human holo transferrin (Prospec)), 100 mM 2-mercaptoethanol, 100 U/ml penicillin, 2mM L-Glutamine, 50 ng/ml SCF, 10 ng/ml IL-3, 10 ng/m IL-6 and 50 ng/ml rmFlt3-ligand (Prospec). Colonies and cells were counted after 7 days of culture at 37°C. For co-culture experiments, LSCs were mixed with 10^4 RPMs from CML mice in transplant medium (see section "CML model") and incubated in 96-well V-bottom plates for 48h prior to culture in methylcellulose.

BrdU incorporation assay

To assess BrdU incorporation in LSCs in the BM and spleen, BrdU was administered intraperitoneally (1mg/mouse) 12 hours before analysis. BrdU staining for flow cytometry analysis was performed using the BD Pharmingen BrdU flow kit (cat. 559619). Briefly, isolated cells were stained with fluorescent surface antibodies, fixed and permeabilized. Cells were then treated with DNase for 60 min at 37°C, stained with BrdU antibody and 7AAD and analyzed using flow cytometry.

***In vivo* imatinib treatment**

Mice were treated 2 x daily with 100mg/kg body weight imatinib mesylate (Novartis) by oral gavage. Control CML mice were treated with water. Treatment was started 4 days after CML induction and continued until the endpoint of the experiment. Eighteen days after CML induction, a spleen/BM ratio of the relative cell frequencies was calculated between imatinib treated and non-treated groups. For secondary transplantation experiments, imatinib and control treated CML mice were euthanized at day 18 after CML induction and total BM and spleen cells were transplanted into lethally irradiated BL/6 recipients. Disease engraftment was subsequently assessed in the peripheral blood.

Imatinib *in vitro* experiments

RPMs were isolated and pooled from 10 BL/6 donor mice. Afterwards, $1x10^4$ RPMs were co-cultured with $1x10^3$ FACS-purified GFP^+ spleen LSCs with and without addition of imatinib (1µM) overnight and plated in MethoCult together with SCF, IL3, IL-6 and Flt3-ligand. Colonies were counted after 7

days. For 2nd platings, colonies were washed with media and $1x10^4$ cells were plated in MethoCult and colonies were counted after 7 days.

Quantitative real time (qRT-) PCR

GFP^+ long-term (lin^- $Sca\text{-}1^+$ $c\text{-}kit^{high}$ $CD150^+$ $CD48^-$) and short-term (lin^- $Sca\text{-}1^+$ $c\text{-}kit^{high}$ $CD150^-$ $CD48^-$) LSCs were isolated from the BM and spleens of pooled CML mice. cDNA was synthesized by using the High-Capacity cDNA Reverse Transcription Kit (Applied Biosystems, USA) and qRT-PCR was performed via FastStart Universal SYBR® Green 2X PCR Master Mix (Roche, Switzerland). Data were clustered using standard Euclidean's method based on the average linkage and heatmaps with Z-scores were generated according to the standard normal distribution of the values.

Silencing of *Ltf* and *S100a9*

For knockdown experiments, GFP^+ BM LSCs of CML mice were sorted and transfected with shRNA lentiviral particles targeting Lactoferrin (*Ltf;* cat. sc-41372-V) and Calgranulin B (*S100a9;* cat. sc-43345-V) or with control shRNA particles (sc-108080) according to the manufacturer's instructions (Santa Cruz Biotechnology). Identical cell numbers were used for colony forming assays and determination of CD11b surface expression. CD11b expression was measured 48h after shRNA tranfection.

Supplementary Figure 1

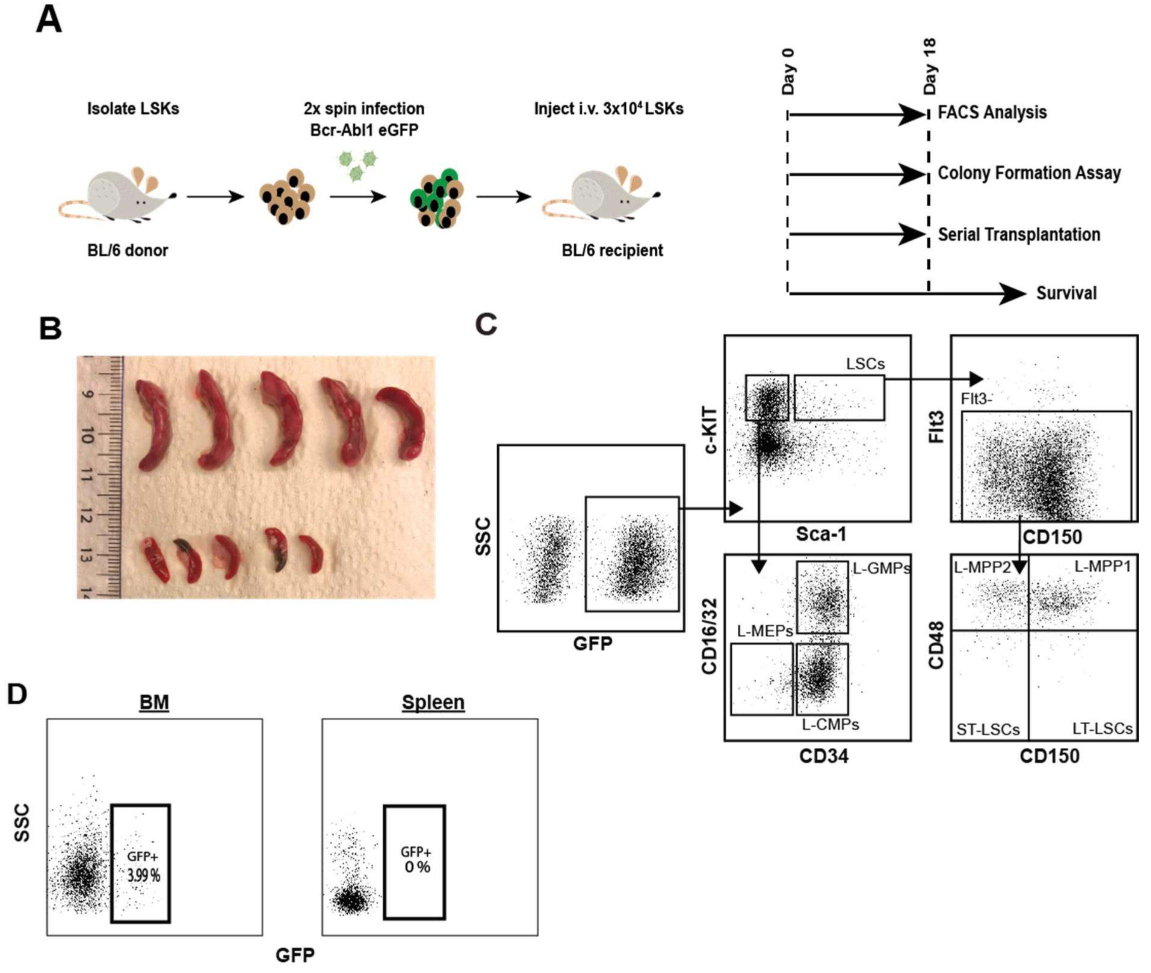

Figure S1: (A) Experimental CML model and time frame. (B) Spleens of CML (upper) and naïve mice (lower). (C) Gating strategy to identify LSPCs. (D) Homing of BCR-ABL1-GFP transduced LSCs. 2 x 10^5 GFP^+ LSCs were i.v. injected into non-irradiated BL/6 mice. Homing of GFP^+, lin^- $ckit^+$,$Sca\text{-}1^+$ LSCs was assessed in BM and spleen 18 hours after transplantation.

Supplementary Figure 2

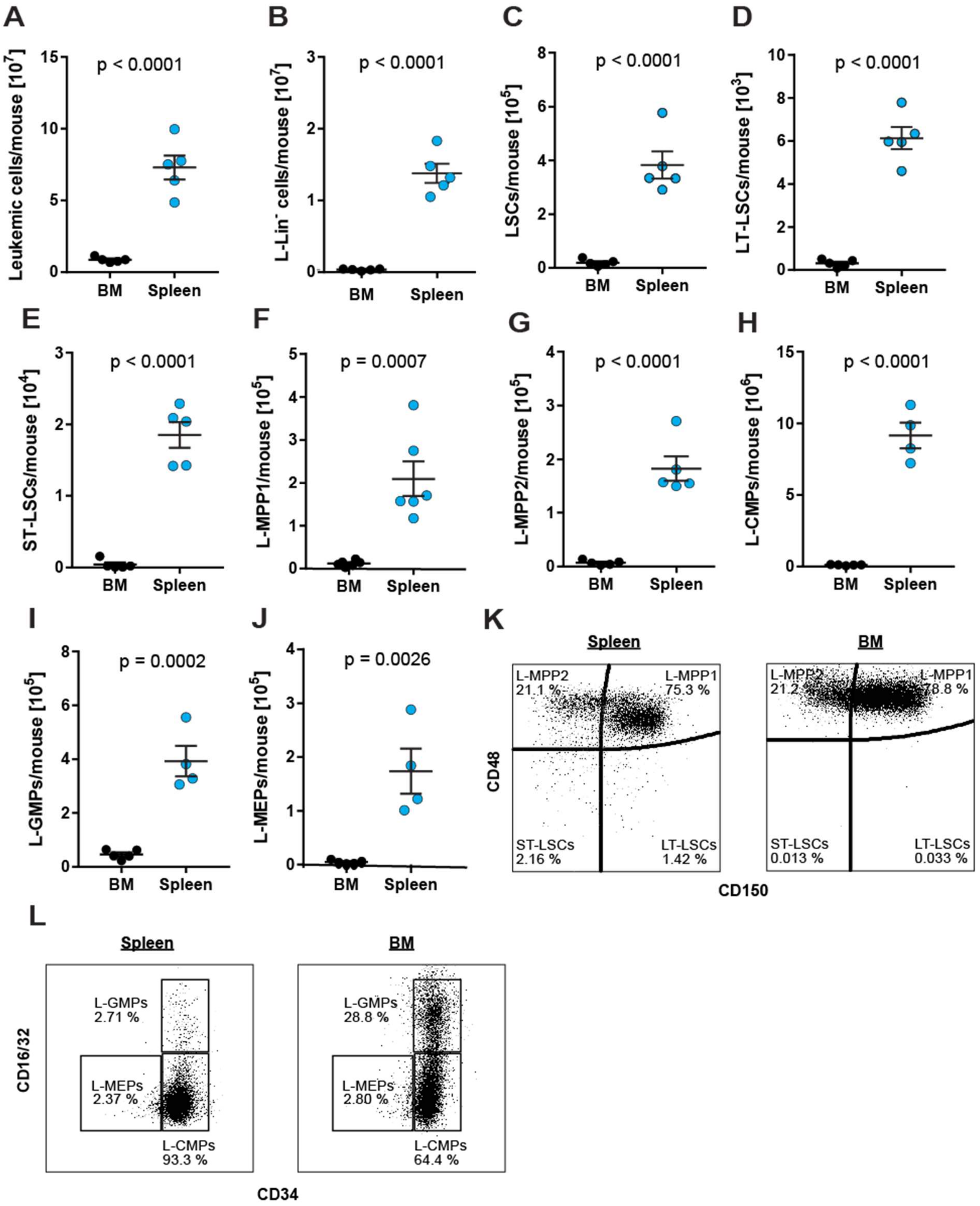

Figure S2. Spleen and BM of CML mice were analyzed 18 days after CML induction. (A) (A-J) Absolute numbers of total leukemic cells, leukemic lineage negative (L-lin$^-$) cells, leukemic stem cells

(LSCs), long-term (LT-) LSCs, short-term (ST-) LSCs, leukemic multipotent progenitors 1 (L-MPP1s), L-MPP2s, leukemic common myeloid progenitors (L-CMPs), leukemic granulocyte-monocyte progenitors (L-GMPs), and leukemic megacaryocyte-erythroid progenitors (L-MEPs). One representative experiment out of 5-10 with n = 4-11 mice per group is shown. Data are displayed as mean ± SEM. Statistics: Unpaired student' t-test.

(K, L) Exemplary FACS-plots of LSPCs.

Supplementary Figure 3

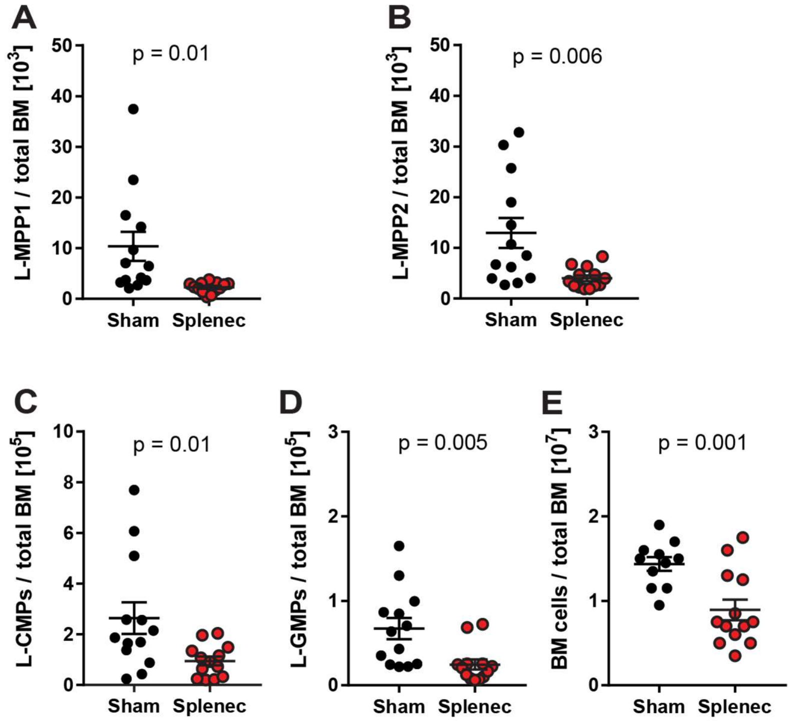

Figure S3. BL/6 mice were splenectomized or sham-operated 14 days prior to CML induction. The leukemic compartment was analyzed 18 days after CML induction in BM. Absolute cell numbers of (A) L-MPP1, (B) L-MPP2, (C) L-CMPs, (D) L-GMPs, and (E) total BM cells. Pooled data from 3 independent experiments with n = 13 mice per group are shown. Data are displayed as mean ± SEM. Statistics: Student’s t-test.

Supplementary Figure 4

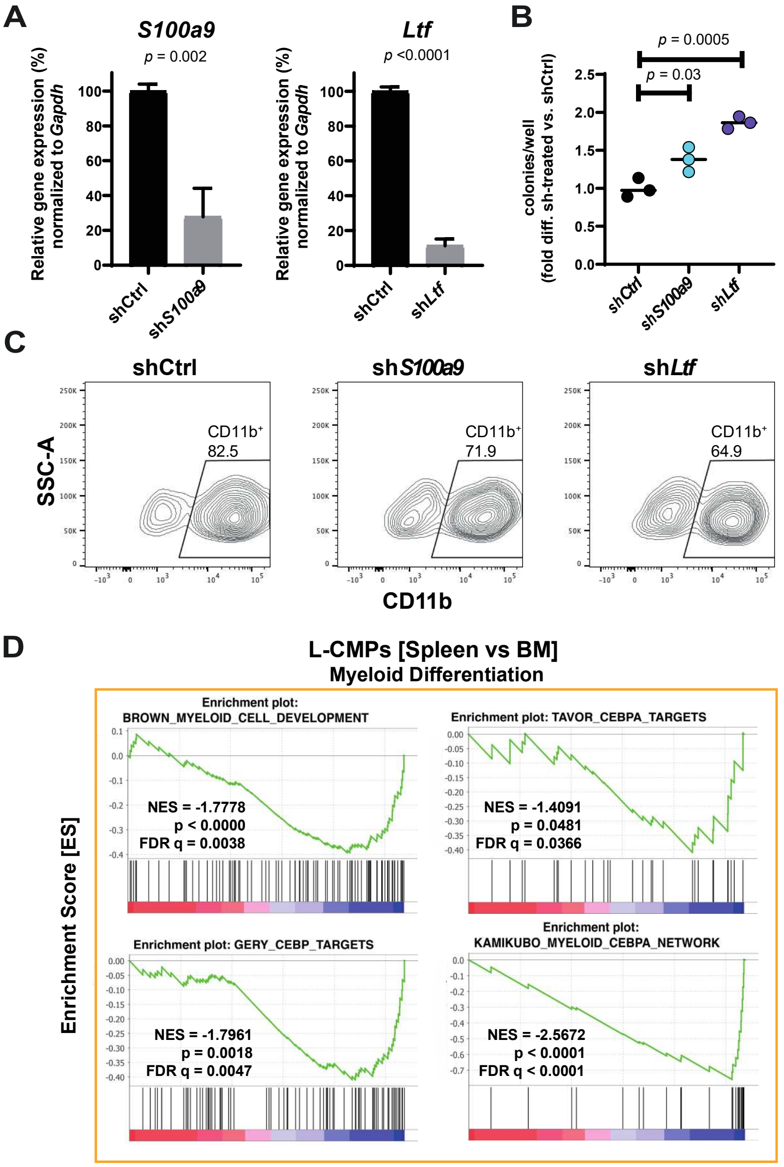

Figure S4. (A) Knockdown efficiency of *shS100a9* and *shLtf* was assessed in LSCs 48 hours after transfection. (B) Colony formation capacity was assessed after 7 days. One representative experiment with n = 3 biological replicates per group is shown. Data are displayed as mean ± SEM. Statistics: Unpaired student's t test (A, B). (C) CD11b was stained and analyzed on LSCs by flow cytometry 24h after knockdown. Percentage of CD11b positive cells is shown. (D) Gene set enrichment analysis (GSEA) representing the normalized enrichment score (NES) and false discovery rate score (FDR) of gene sets linked to myeloid differentiation for L-CMPs (spleen vs. BM).

Supplementary Figure 5

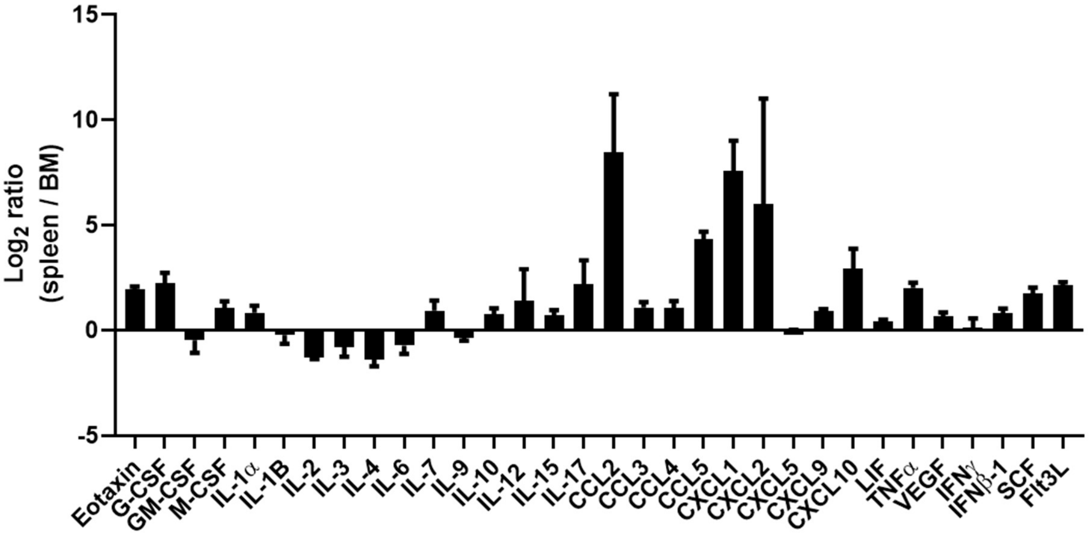

Figure S5. Analysis of spleen and BM supernatants of CML mice. Histogram indicating ratio of the concentration of listed cytokines, chemokines and growth factors in spleen vs. BM. N = 3 biological replicates with 5 pooled mice per replicate.

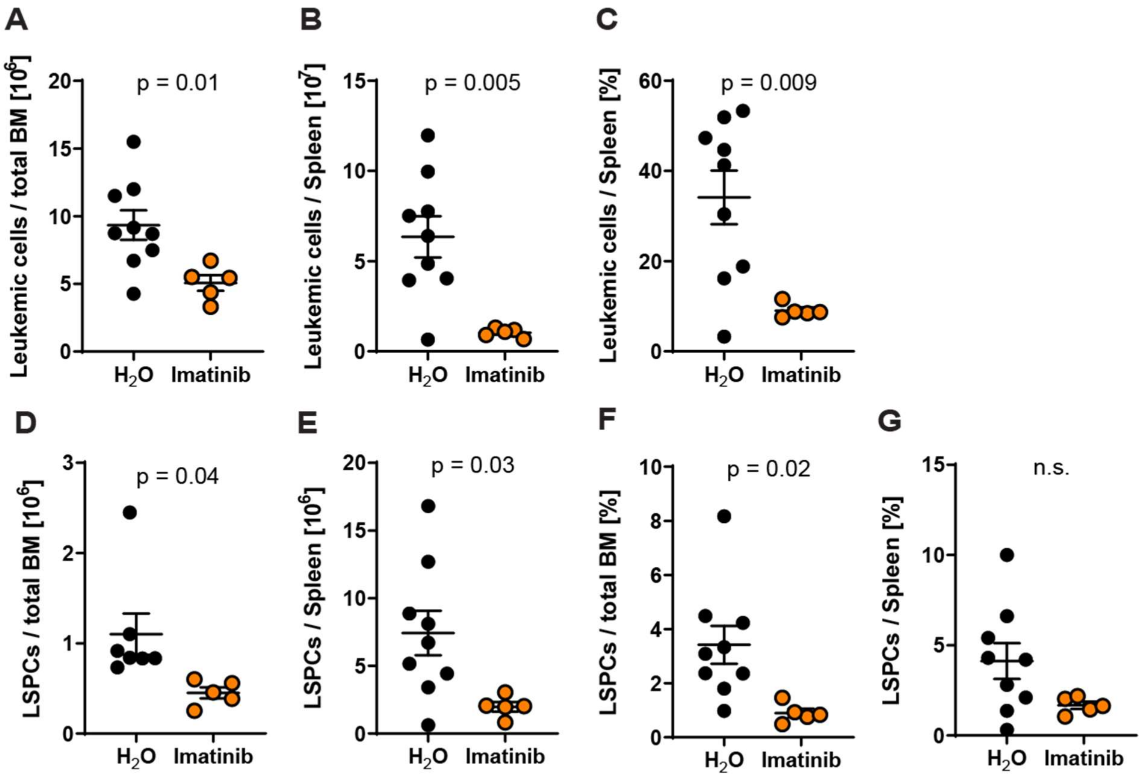

Figure S6. Absolute cell numbers of total leukemic cells in (A) BM and (B) spleen. (C) Frequencies of total leukemic cells in the Spleen. Absolute cell numbers of LSPCs in (D) BM and (E) spleen. Frequencies of LSPC in (F) BM and (G) spleen. Pooled data from 2 independent experiments with n = 5-9 mice per group are shown. Data are displayed as mean ± SEM. Statistics: Student's t-test.

Supplementary Figure 7

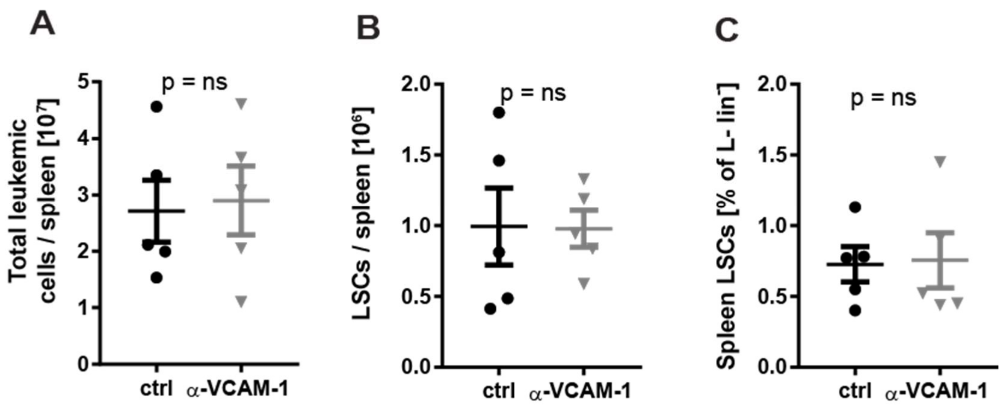

Figure 7. BL/6 CML mice were treated with VCAM-1 blocking antibody or IgG control. Spleens were analyzed 18 days after CML induction. (A) Total leukemic spleen cells. (B) Absolute and (C) relative LSC numbers in the spleen. 1 out of 2 independent experiment with n = 5 mice is shown. Data are displayed as mean ± SEM. Statistics: Student’s t-test.

Supplementary Figure 8

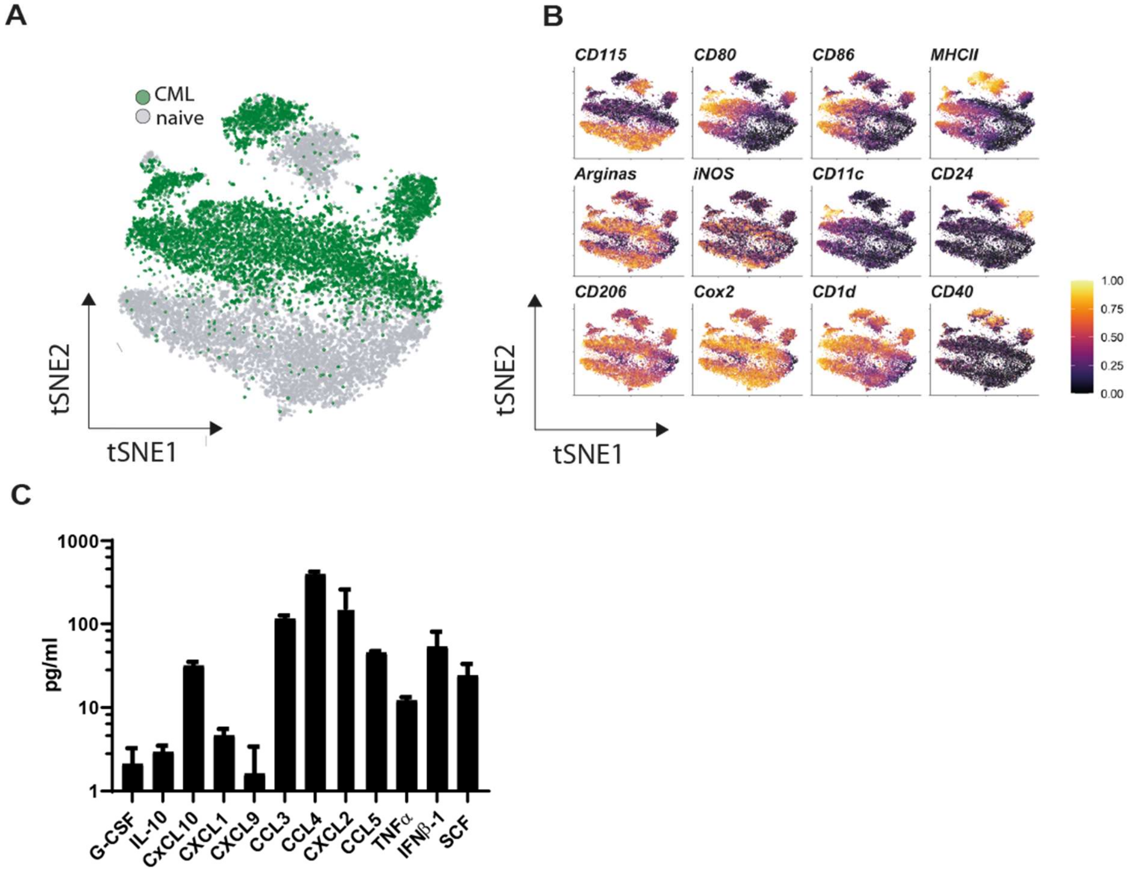

Figure S8. (A) Clustering after CyTOF analysis and dot plot representation of tSNE analysis in RPMs of naïve and CML mice. (B) Dot plot representation of tSNE analysis. One representative experiment with n =20 (CML) and n = 10 (naïve) pooled mice. (C) Cytokines and chemokines produced by in-vitro cultured RPMs. One representative experiment with n = 3 biological replicates.

Supplementary Table 1

Surface staining

Target	Company	Catalog number	Clone	Mass	Metal
Cx43	ThermoFisher	13-8300	Cx-1B1	143	Nd
CD64	Fluidigm	3151012B	X545/7.1	151	Eu
CD196	Fluidigm	3156016A	292L17	156	Gd
CD93	Fluidigm	3158015B	AA4.1	158	Gd
CD317	Biolegend	127002	927	159	Tb
CD86	Biolegend	105102	PO3	163	Dy
CX3CR1	Biolegend	149002	SA011F11	167	Er
CD206	Fluidigm	3169021B	C068C2	169	Tm
CD80	Fluidigm	3171008B	1610A1	171	Yb
CD117	Fluidigm	3173004B	2B8	173	Yb
CD192	R&D	MAB55381-100	475301	176	Yb
F4/80	Fluidigm	3146008B	BM8	146	Nd
CD274	Fluidigm	3153016B	10F.9G2	153	Eu
CD40	Biolegend	102902	HM40-3	154	Sm
CD49b/DX5	Fluidigm	3164011B	HMa2	164	Dy
CD11c	Fluidigm	3142003B	N418	142	Nd
CD11b	Fluidigm	3148003B	M1/70	148	Nd
CD19	Fluidigm	3149002B	6D5	149	Sm
CD24	Fluidigm	3150009B	M1/69	150	Nd
CD3e	Fluidigm	3152004B	145-2C11	152	Sm
CD1d	Fluidigm	3162020B	1B1	162	Dy
CD161	Fluidigm	3170002B	PK136	170	Er
I-A/I-E	Fluidigm	3174003B	M5/114.15.2	174	Yb
Ly6G	Fluidigm	3141008B	1A8	141	Pr
CD115	Fluidigm	3144012B	AFS98	144	Nd
CD8a	Fluidigm	3168003B	53-6.7	168	Er
CD45	Fluidigm	3147003B	30-F11	147	Sm
CD4	Fluidigm	3172003B	RM45	172	Yb

Cytoplasmic/nuclear staining

Target	Company	Catalog number	Clone	Mass	Metal
iNOS	eBioscience	14-5920-82	CXNFT	155	Gd
Arginase-1	Fluidigm	3166023B	polyclonal	166	Er
Cox2	R&D	AF4798	polyclonal	160	Gd
Gata6	Fluidigm	3161005A	D61E4	161	Dy
PKM2	proteintech	PA5-25962	polyclonal	165	Ho

Supplementary Table 2

Patient characteristics

Case #	Age at diagnosis (y)	Sex	Year of diagnosis	Pathological diagnosis	Spleen weight (g)
1	53	m	1990	CML	3520
2	71	m	1993	CML	3000
3	57	m	1997	CML	1800
4	34	f	2002	CML	1270
